# D-Transposition of the great arteries with restrictive foramen ovale in the fetus: the dilemma of predicting the need for postnatal urgent balloon atrial septostomy

**DOI:** 10.1007/s00404-023-06997-8

**Published:** 2023-03-27

**Authors:** I. Gottschalk, A. Walter, T. Menzel, E. C. Weber, S. Wendt, N. Sreeram, U. Gembruch, C. Berg, J. S. Abel

**Affiliations:** 1 Division of Prenatal Medicine, Gynecological Ultrasound and Fetal Surgery, Department of Obstetrics and Gynecology, University Hospital Cologne and Faculty of Medicine, University of Cologne, Cologne, Germany; 2 Department of Obstetrics and Prenatal Medicine, University of Bonn, Bonn, Germany; 3 Heartcenter, Department of Cardiac Surgery, Cardiothoracic Intensive Care and Thoracic Surgery, University Hospital Cologne, University of Cologne, Cologne, Germany; 4 Department of Pediatric Cardiology, University Hospital Cologne and Faculty of Medicine, University of Cologne, Cologne, Germany

**Keywords:** Prenatal, Ultrasound, TGA, Fetus, Restrictive foramen ovale, Balloon atrial septostomy

## Abstract

**Objective:**

Restrictive foramen ovale (FO) in dextro-transposition of the great arteries (d-TGA) with intact ventricular septum may lead to severe life-threatening hypoxia within the first hours of life, making urgent balloon atrial septostomy (BAS) inevitable. Reliable prenatal prediction of restrictive FO is crucial in these cases. However, current prenatal echocardiographic markers show low predictive value, and prenatal prediction often fails with fatal consequences for a subset of newborns. In this study, we described our experience and aimed to identify reliable predictive markers for BAS.

**Methods:**

We included 45 fetuses with isolated d-TGA that were diagnosed and delivered between 2010 and 2022 in two large German tertiary referral centers. Inclusion criteria were the availability of former prenatal ultrasound reports, of stored echocardiographic videos and still images, which had to be obtained within the last 14 days prior to delivery and that were of sufficient quality for retrospective re-analysis. Cardiac parameters were retrospectively assessed and their predictive value was evaluated.

**Results:**

Among the 45 included fetuses with d-TGA, 22 neonates had restrictive FO postnatally and required urgent BAS within the first 24 h of life. In contrast, 23 neonates had normal FO anatomy, but 4 of them unexpectedly showed inadequate interatrial mixing despite their normal FO anatomy, rapidly developed hypoxia and also required urgent BAS (‘bad mixer’). Overall, 26 (58%) neonates required urgent BAS, whereas 19 (42%) achieved good O_2_ saturation and did not undergo urgent BAS. In the former prenatal ultrasound reports, restrictive FO with subsequent urgent BAS was correctly predicted in 11 of 22 cases (50% sensitivity), whereas a normal FO anatomy was correctly predicted in 19 of 23 cases (83% specificity). After current re-analysis of the stored videos and images, we identified three highly significant markers for restrictive FO: a FO diameter < 7 mm (*p* < 0.01), a fixed (*p* = 0.035) and a hypermobile (*p* = 0.014) FO flap. The maximum systolic flow velocities in the pulmonary veins were also significantly increased in restrictive FO (*p* = 0.021), but no cut-off value to reliably predict restrictive FO could be identified. If the above markers are applied, all 22 cases with restrictive FO and all 23 cases with normal FO anatomy could correctly be predicted (100% positive predictive value). Correct prediction of urgent BAS also succeeded in all 22 cases with restrictive FO (100% PPV), but naturally failed in 4 of the 23 cases with correctly predicted normal FO (‘bad mixer’) (82.6% negative predictive value).

**Conclusion:**

Precise assessment of FO size and FO flap motility allows a reliable prenatal prediction of both restrictive and normal FO anatomy postnatally. Prediction of likelihood of urgent BAS also succeeds reliably in all fetuses with restrictive FO, but identification of the small subset of fetuses that also requires urgent BAS despite their normal FO anatomy fails, because the ability of sufficient postnatal interatrial mixing cannot be predicted prenatally. Therefore, all fetuses with prenatally diagnosed d-TGA should always be delivered in a tertiary center with cardiac catheter stand-by, allowing BAS within the first 24 h after birth, regardless of their predicted FO anatomy.

## What does this study add to the clinical work

**Table:** 

Restrictive foramen ovale (FO) in dextro-transposition of the great arteries (d-TGA) with intact ventricular septum may lead to severe life-threatening hypoxia within the first hours of life, making urgent balloon atrial septostomy (BAS) inevitable. Precise assessment of FO size and FO flap motility is possible prenatally and allows a reliable prediction of both restrictive and normal FO anatomy postnatally. In addition, prediction of likelihood of urgent BAS also succeeds reliably in all fetuses with restrictive FO, but identification of the small subset of fetuses that also requires urgent BAS despite their normal FO anatomy fails. Therefore, all fetuses with prenatally diagnosed d-TGA should always be delivered in a tertiary center with cardiac catheter stand-by, allowing BAS within the first 24 hours after birth, regardless of their predicted FO anatomy.	

## Introduction

The simple dextro-Transposition of the Great Arteries with intact ventricular septum (d-TGA) is one of the most common cyanotic heart defects with a prevalence of 2–3 of 10.000 live births. It accounts for 5–7% of all congenital heart defects [1, 2]. Simple d-TGA is characterized by atrioventricular concordance and ventriculo-arterial discordance, leading to redistribution of blood flow with subsequent development of severe hypoxemia and rapid hemodynamic compromise postnatally. Both, fetal pulmonary circulation and ductus arteriosus become increasingly sensitive to the high oxygen content toward the end of gestation, which may predispose to a restriction of the foramen ovale (FO) and ductus arteriosus at term by increase of left atrial pressure leading to restrictive FO flow [3–7]. In addition, the increased right ventricular outflow and decreased antegrade flow through the arterial duct lead to a reduced right-to-left shunt across the FO and to a stagnation of FO growth during the 3rd trimester [8, 9]. Consequently, the FO may be significantly restrictive in a significant proportion of newborns, making an urgent balloon atrial septostomy (BAS) immediately after birth inevitable in about 28% of newborns [9, 10]. Indisputably, prenatal diagnosis of critical d-TGA with restrictive FO with planned delivery in a tertiary center with cardiac catheter stand-by, allowing immediate BAS after birth, improves neonatal survival, whereas a significant delay of diagnosis and intervention postnatally may result in severe life-threatening neonatal hypoxia [11–13].

Several conventional prenatal echocardiographic parameters were previously evaluated with contradictory sensitivity and specificity in predicting restrictive FO and the likelihood for urgent BAS [6, 7, 9, 14–21]. Recently, modern techniques on cardiac functional assessment like spectral pulsed wave tissue-Doppler imaging, speckle-tracking echocardiography, or fetal cardiac magnetic resonance imaging have demonstrated a potentially higher sensitivity for detection of subtle subclinical changes in myocardial dysfunction [7, 22–24], but data on functional assessment in fetuses with d-TGA are limited to only one single small cohort of only 13 fetuses without validation in larger prospective trials [7]. Additionally, the calculation of functional cardiac indices is time-consuming and requires highly specified instrumental equipment. Therefore, the use of these technologies is so far limited to a very few specialized centers and research purposes only.

This study aimed to describe our experience with the prenatal prediction of normal or restrictive FO anatomy in fetuses with d-TGA. We first reviewed the reliability of our own former prenatal ultrasound diagnoses made at the time of the patients’ visits in our centers prior to birth. Second, we re-assessed all stored echocardiographic videos and still images and correlated them with the outcome. Thereby, we retrospectively identified the most reliable echocardiographic parameters for restrictive FO. Finally, we described the predictive value of these parameters by calculating their sensitivity and specificity.

## Methods

### Study design

We conducted a retrospective cohort study in two large German tertiary referral centers (University Hospitals of Cologne and Bonn) over a period of 13 years (2010–2022) and included all cases of isolated fetal d-TGA with intact ventricular septum and known outcome. Requirements for inclusion in the study were the availability of the former prenatal ultrasound reports as well as sufficient prenatal echocardiographic videos and still images for re-evaluation and to establish prognostic markers. We only included cases where last echocardiographic examination was performed not more than 14 days prior to delivery. Exclusion criteria were d-TGA with hemodynamically relevant ventricular septal defects, presence of additional major anomalies, delivery before completed 34 weeks, lack of sufficient prenatal echocardiographic videos, and images for retrospective re-evaluation as well as unknown outcome.

The fetal and maternal variables assessed in every case are shown in Table 1. Prenatal testing for aneuploidies including 22q11 microdeletion was offered to all parents. The assessment of postnatal outcome included gestational age at delivery, mode of delivery, birth weight, Apgar score at 1 min, oxygen saturation, need for urgent BAS within the first 24 h after birth, and day of life at arterial switch operation. All neonatal data and clinical files were retrieved from our Departments of Pediatric Cardiology in Cologne and Bonn.

**Table 1:** Fetal and maternal characteristics in the urgent and no urgent BAS group

Characteristics	All	Urgent BAS	No urgent BAS	*p* value
	(*n* = 45)	(*n* = 26)	(*n* = 19)	
Maternal age (years)	32.6 ± 4.8	32.5 ± 4.7	32.6 ± 5.0	0.934
Gestational age at last echo (weeks)	38 + 6 (34 + 5–40 + 1)	38 + 4 (37 + 1–39 + 1)	38 + 1 (34 + 5–40 + 2)	0.368
Estimated fetal weight at last ultrasound (g)	3133 ± 665	3290 ± 650	3240 ± 677	0.505
Period between last echocardiography—birth	7.9 ± 4.5	7.2 ± 3,4	8.3 ± 4.4	0.544
Gestational age at delivery (weeks)	38 + 6 (35 + 0–40 + 6)	38 + 6 (37 + 6–40 + 1)	38 + 4 (35 + 0–40 + 6)	0.467
Cesarean section	26 (57.8%)	16 (61.5%)	10 (52.6%)	0.550
Birth weight (g)	3400 (range 2500–4100)	3390 (range 2500–4100)	3450 (range 2700–4000)	0.848
Age of life at arterial switch operation (days)	9 (1–30)	8 (1–16)	10 (4–30)	0.600

Retrospective studies were approved by the local ethical committee of human research (No 20-1517).

### Predictive reliability of the former prenatal ultrasound reports

For the purpose of this study, ultrasound reports were retrieved from the ViewPoint Software system (GE Healthcare) and compared with the outcome to retrospectively evaluate the predictive reliability of the FO anatomy. At that time, prenatal sonographic assessment as well as prediction of the likelihood of urgent BAS was performed by varying operators and according to subjective criteria.

### Establishing reliable diagnostic cardiac parameters

The cardiac parameters, that were retrospectively calculated to establish reliable prognostic parameters, were retrieved from the latest echocardiographic videos and still images that were recorded not more than 2 weeks prior to delivery. This retrospective calculation was always carried out by the same author (I.G.) and blinded to the outcome. Then, the author once again predicted the likelihood for urgent BAS and the specificity and sensitivity of this current assessment was described.

**Fig. 1:**
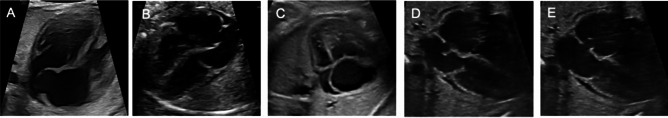
Sonographic appearance of the FO flap. **A** redundant FO flap that extents more than 50% across the left atrium (‘spinnaker-like’, **B** flat FO, the flap swings less than 30% into the left atrium, **C** fixed FO without any swinging movements of the flap, **D** and **E** hypermobile FO flap, that bidirectionally oscillates through the small FO orifice between the left (**D**) and the right atrium (**E**)

The cardiac parameters that were assessed, are shown in Table 2. We measured both the right and left atrial diameter in the 4-chamber view and calculated the right-to-left atrial diameter ratio as previously described by Firpo et al. [25]. We assessed the sonographic appearance of the septum primum and the FO flap swinging movement and measured the diameter of the FO as well as the maximal angle of the flap at its attachment point to the septum which usually varies between 30 and 50° as described by Wilson et al. [26]. In case of an abnormal mobility of the FO flap, we described the FO flap either as (a) redundant, if the aneurysmal septum primum flap bulged more than 50% across the left atrium wall (‘spinnaker-like’), (b) flat, if the angle between the septum primum flap and the rest of the atrial septum was < 30°, (c) fixed, if the septum primum did not demonstrate any swinging mode during cardiac cycle, or (d) hypermobile, if the flap oscillated bidirectionally through the small FO orifice between the left and right atrium (Fig. 1).

**Table 2:** Retrospectively calculated cardiac parameters

Cardiac parameters	All	Urgent BAS	No urgent BAS	*p* value
	(*n* = 45)	(*n* = 26)	(*n* = 19)	
RA diameter (mm)	22.61 ± 3.46	22.86 ± 3.02	22.11 ± 2.85	0.428
LA diameter (mm)	20.65 ± 2.89	20.86 ± 3.02	20.38 ± 2.78	0.595
RA-to-LA diameter ratio, mean ± SD	1.12 ± 0.12	1.12 ± 0.07	1.11 ± 0.19	0.317
FO diameter (mm)	7.05 ± 2.25	5.71 ± 2.06	8.75 ± 0.95	< 0.01*
FO restrictive appearance (< 7 mm) (*n*)	20 (44.4%)	20 (76.9%)	0 (0%)	< 0.01*
Redundant ‘spinnaker-like’ appearance of the FO flap (*n*)	4 (8.9%)	3 (11.5%)	1 (5.3%)	0.465
Flat appearance of the FO flap (mobility < 30°) (*n*)	4 (8.9%)	3 (11.5%)	1 (5.3%)	0.465
Fixed appearance of the FO flap (*n*)	9 (20.0%)	8 (30.8%)	1 (5.3%)	0.035*
Hypermobile FO flap (*n*)	7 (15.6%)	7 (26.9%)	0 (0%)	0.014*
‘Plateau-like’ PV flow pattern (*n*)	24/28 (85.7%)	16/18 (88.9%)	8/10 (80%)	0.520
‘pulsatile’ PV flow pattern (*n*)	2/28 (7.1%)	2/18 (11.1%)	2/10 (20%)	0.520
To-and-fro PV flow pattern (*n*)	2/28 (7.1%)	2/18 (11.1%)	0/10 (0%)	0.274
PV maximum systolic flow velocity (cm/s), mean ± SD	34 ± 8	35 + / 9	31 ± 6	0.021*
PV maximum systolic flow velocity ≥ 35 cm/s (*n*)	15/28 (53.6%)	11/18 (61.1%)	4/10 (40%)	0.062
PV maximum systolic flow velocity ≥ 41 cm/s (*n*)	4/28 (14.3%)	4/18 (22.2%)	0/10 (0%)	0.107
Restrictive DA (*n*)	2 (4.4%)	2 (7.7%)	0 (0%)	0.216
Reversed flow in DA (*n*)	2 (4.4%)	2 (7.7%)	0 (0%)	0.216

**Fig. 2:**
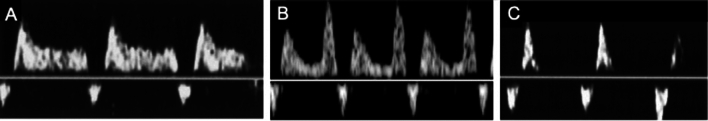
Flow patterns of pulmonary veins. **A** continuous forward flow with a ‘plateau’ and small a-wave reversal, **B** continuous forward flow with an increased a-wave reversal (‘pulsatile’), **C** to-and-fro flow pattern with absent early diastolic forward flow, highly suspicious for restrictive FO

The Doppler flow patterns in the pulmonary veins were classified either as (a) continuous forward flow with a small a-wave reversal (‘plateau-like’ flow pattern), (b) continuous forward flow with an increased a-wave reversal (‘pulsatile’ flow pattern) or (c) to-and-fro flow pattern with absent diastolic forward flow. In accordance with Taketazu et al. [27] we classified the ‘plateau-like’ and the ‘pulsatile’ flow patterns as not suspicious and the to-and-fro flow pattern as highly suspicious for restrictive FO (Fig. 2).

**Fig. 3:**
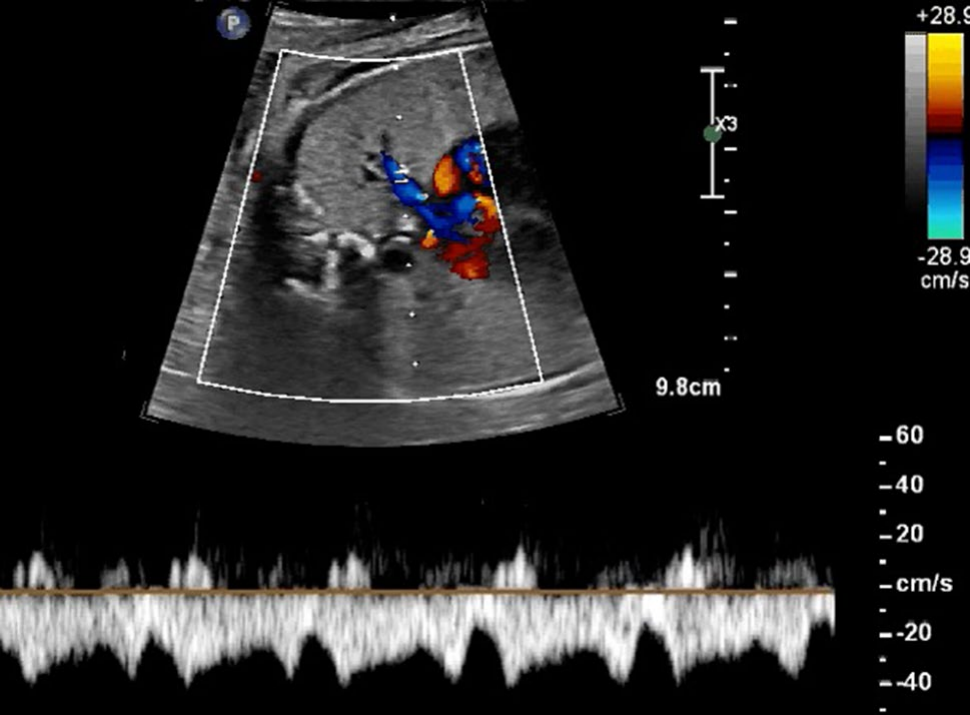
Maximum Doppler flow velocity in the pulmonary vein

The Doppler flow velocity was measured in at least one pulmonary vein at the entrance to the left atrium as described by Slodki et al. [19], and the maximum systolic flow velocity was assessed (Fig. 3). The angle of insonation was preferably 0°, but always less than 30°. As previously described, a maximum systolic flow velocity of ≥ 35 cm/s was classified as increased and ≥ 41 cm/s as highly suspicious for restrictive FO.

**Fig. 4:**
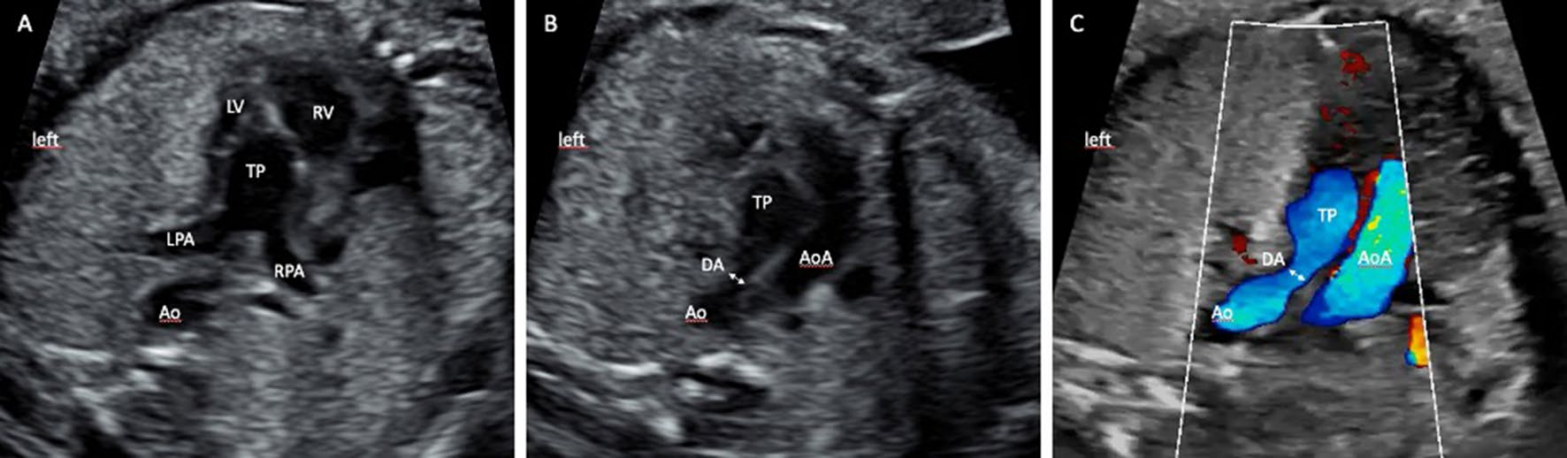
DA in the 3-vessel view in B-mode and Color Doppler. **A** ventriculo-arterial discordance with the pulmonary trunk arising from the left ventricle, **B** 3-vessel view with restrictive arterial duct, the arrow shows the size of the arterial duct, **C** 3-vessel view in Color Doppler, showing an antegrade perfusion of the restrictive arterial duct. (*LV* left ventricle, *RV* right ventricle, *TP* main pulmonary trunk, *LPA* left pulmonary artery, *RPA* right pulmonary artery, *Ao* descending aorta, *AoA* aortic arch)

The size of the ductus arteriosus (DA) and the direction of flow in the DA were routinely assessed in all examinations in the 3-vessel view by B-mode and Color Doppler, respectively (Fig. 4). A reversed flow in the DA was classified as abnormal. Atrial diameters, FO diameter, FO flap appearance and flap mobility, ductal size, and flow direction in arterial duct could be assessed in all fetuses, whereas the pulmonary vein (PV) flow patterns and the PV systolic maximum flow velocity could retrospectively be calculated in only 28 of our 45 fetuses.

### Standardized postnatal management

Postnatal management in both of our participating institutes was standardized. A cardiological team with ultrasound system and full catheterization equipment was on stand-by as soon as the birth was about to begin, so that a BAS could be performed immediately after birth, if necessary. In both institutes, we recommended delivery by planned cesarean section in all cases with prenatally suspected restrictive FO, whereas in cases of a predicted normal FO anatomy, the mode of delivery was chosen by the obstetric condition or by maternal request. Immediately after birth, a focused echocardiography was performed to first ascertain the prenatal diagnosis of d-TGA with intact ventricular septum and second to evaluate the FO anatomy and the interatrial shunt. Initial treatment included administration of prostaglandin E_1_, intubation, and ventilation on the basis of the clinical status of the neonate. Urgent BAS was performed within the first 24 h of life in all neonates with a severely restrictive FO and persistent neonatal hypoxemia at first assessment with oxygen saturation of ≤ 65%. If oxygen saturation was normal despite reduced FO diameter, BAS was not considered as urgent and was not performed within the first 24 h.

### Statistical analysis

Statistical analysis was performed using Student’s *t* Test for continuous variables and the *χ*^2^ or Fisher’s exact test for categorical variables. A *p *value of < 0.05 was considered statistically significant, and all *p*-values were based upon two-tailed tests. Statistical analysis was performed using SPSS for Mac (SPSS Inc., Chicago, Ill., USA).

## Results

During the study period, 87 fetuses with isolated d-TGA with intact ventricular septum and known outcome were diagnosed and delivered in one of our two participating centers. 42 cases had to be excluded from the study. Reasons for exclusion were either preterm delivery before the completed 34th week or lack of suitable prenatal echocardiographic videos or still images for reliable re-analysis. Finally, 45 cases were included in this series. Fetal or neonatal genetic testing, including karyotype and 22q11 microdeletion, was normal in all cases. There was no termination of pregnancy and no intrauterine or neonatal death in our cohort.

Cesarean section was performed in 26 (57.7%) of 45 cases. Reasons for surgical delivery were either prenatal prediction of restrictive FO in 15 cases, obstetrical reasons in seven cases and maternal request in four cases. Median gestational age at delivery was 39 weeks (range, 36–41 weeks).

**Fig. 5:**
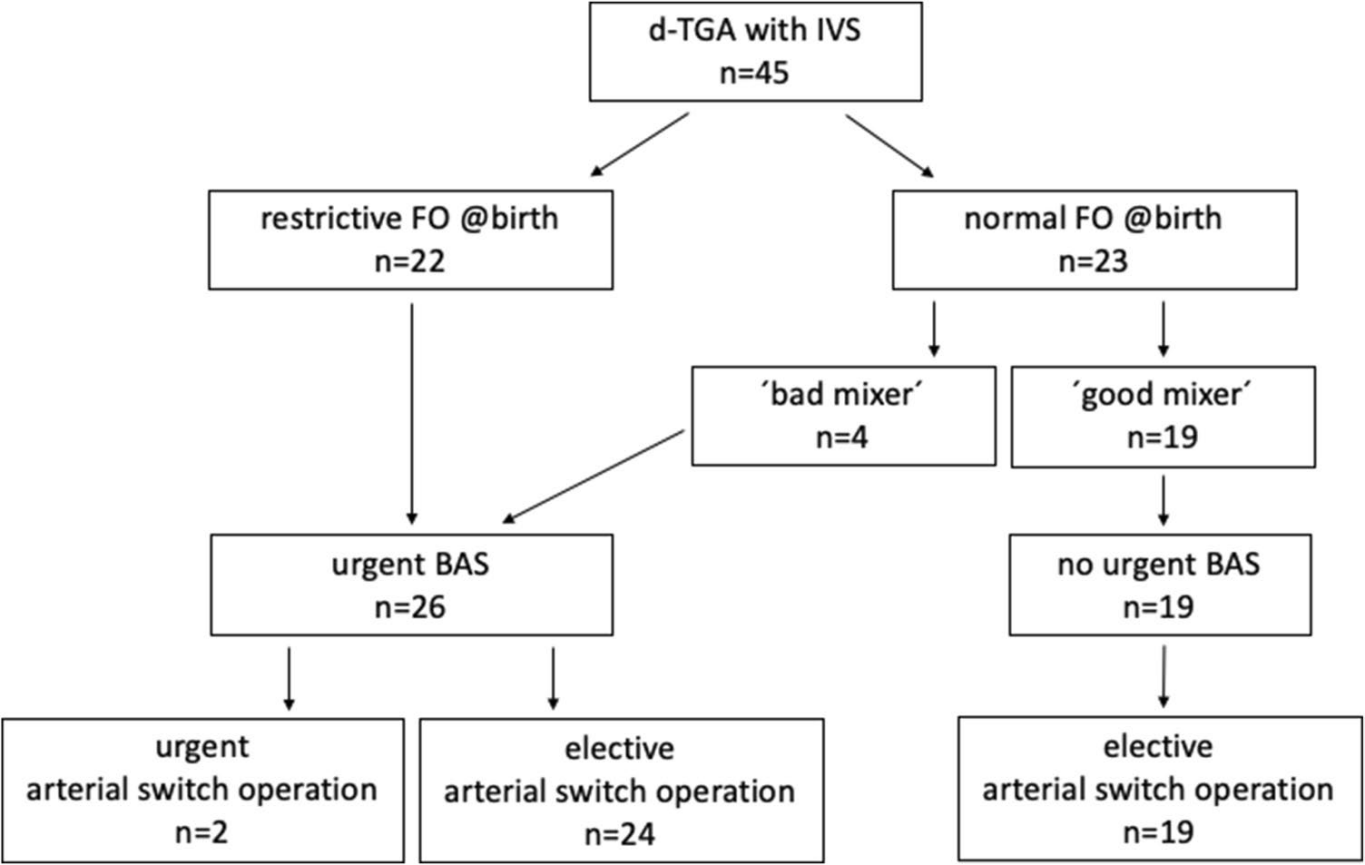
Flow chart of 45 neonates with d-TGA (*IVS* intact ventricular septum, *FO* foramen ovale, *BAS* balloon atrial septostomy)

Among the 45 fetuses, 22 neonates had restricted FO at birth and underwent urgent BAS within the first 24 h of life, whereas 23 neonates had normal FO size at birth. Among the latter, four neonates unexpectedly showed inadequate interatrial mixing despite a normal FO anatomy. They did not achieve sufficient oxygen saturation, rapidly developed hypoxia, and also required urgent BAS (‘bad mixer’). Finally, 26 (57.8%) neonates required urgent BAS within the first 24 h of life (urgent BAS group), whereas 19 (42.2%) neonates achieved good oxygen saturation and did not underwent urgent BAS (non-urgent BAS group) (Fig. 5).

Among the 4 “bad mixers”, all had normal FO appearance with normal FO diameters between 8 and 9 mm, all had a normal FO flap mobility of 30–50° and none of them had a restrictive DA prenatally. Additionally, in 3 of those 4 cases, the pulmonary veins were also assessed prenatally and also showed a normal, plateau-like flow pattern with peak velocities between 20 and 30 cm/s (Table 3, patients no 23–26). All those prenatal findings were confirmed on first postnatal echocardiography that was performed immediately after birth.

**Table 3:** Prediction of FO anatomy and likelihood of urgent BAS in the former ultrasound reports and after retrospective re-analysis of 26 fetuses that required urgent BAS

Patient no	Former prenatal ultrasound report	Retrospective re-analysis of FO assessment	Retrospective re-analysis of FO diameter (mm)	Retrospective re-analysis of FO flap movement	Retrospective re-analysis of PV flow pattern	Retrospective re-analysis of PV peak velocity(cm/s)	Retrospective re-analysis of DA flow direction	Delivery Mode	Birth weight (kg)	Postnatal findings	Urgent BAS
1	Normal	Restrictive	10	Flat	Plateau	43	Antegr	Cesarean	2500	fixed flap	Yes
2	Normal	Restrictive	5	Fixed	To-and-fro	37	Antegr	Cesarean	3100	FO < 7 mm, fixed flap	Yes
3	Normal	Restrictive	5	Hypermobile	Plateau	38	Antegr	Cesarean	3400	FO < 7 mm, hypermobile flap	Yes
4	Restrictive	Restrictive	6	Fixed	Plateau	30	Antegr	Cesarean	3600	FO < 7 mm, fixed flap	Yes
5	Normal	Restrictive	3	Hypermobile	Plateau	20	Antegr	Vaginal	3800	FO < 7 mm, hypermobile flap	Yes
6	Restrictive	Restrictive	4	Fixed	To-and-fro	42	Antegr	Cesarean	3000	FO < 7 mm, fixed flap	Yes
7	Restrictive	Restrictive	10	Redundant	Plateau	43	Antegr	Cesarean	4000	Fixed flap	Yes
8	Normal	Restrictive	6	Hypermobile	Plateau	38	Antegr	Vaginal	3800	FO < 7 mm	Yes
9	Normal	Restrictive	5	Redundant	Plateau	29	Reversed	Vaginal	3600	FO < 7 mm, hypermobile flap	Yes
10	Restrictive	Restrictive	4	Flat	Plateau	35	Reversed	Cesarean	3100	FO < 7 mm, fixed flap	Yes
11	Restrictive	Restrictive	4	Fixed	Plateau	35	Antegr	Cesarean	3200	FO < 7 mm, fixed flap	Yes
12	Normal	Restrictive	4	Redundant	Plateau	58	Antegr	Cesarean	2700	FO < 7 mm	Yes
13	Restrictive	Restrictive	4	Hypermobile	Plateau	35	Antegr	Cesarean	3900	FO < 7 mm, hypermobile flap	Yes
14	Restrictive	Restrictive	3	Hypermobile	Plateau	40	Antegr	Cesarean	3700	FO < 7 mm, hypermobile flap	Yes
15	Restrictive	Restrictive	4	Flat	Plateau	28	Antegr	Cesarean	4000	FO < 7 mm, flat flap	Yes
16	Normal	Restrictive	5	Normal			Antegr	Vaginal	3400	FO < 7 mm	Yes
17	Normal	Restrictive	5	Fixed			Antegr	Vaginal	3500	FO < 7 mm, fixed flap	Yes
18	Normal	Restrictive	5	Fixed			Antegr	Cesarean	3000	FO < 7 mm, fixed flap	Yes
19	Restrictive	Restrictive	3	Hypermobile			Antegr	Cesarean	3700	FO < 7 mm, hypermobile flap	Yes
20	Normal	Restrictive	5	Hypermobile			Antegr	Vaginal	3300	FO < 7 mm	Yes
21	Restrictive	Restrictive	5	Fixed			Antegr	Cesarean	4100	FO < 7 mm, fixed flap	Yes
22	Restrictive	Restrictive	6	Fixed			Antegr	Cesarean	3100	FO < 7 mm, fixed flap	Yes
23	Normal	Normal	8	Normal	Plateau	30	Antegr	Vaginal	3100	FO > 7 mm, normal flap motility, but bad mixer	Yes
24	Normal	Normal	8	Normal	Plateau	30	Antegr	Vaginal	2900	FO > 7 mm, normal flap motility, but bad mixer	Yes
25	Normal	Normal	9	Normal	Plateau	20	Antegr	Vaginal	3600	FO > 7 mm, normal flap motility, but bad mixer	Yes
26	Normal	Normal	8	Normal			Antegr	Vaginal	3000	FO > 7 mm, normal flap motility, but bad mixer	Yes

### Urgent BAS group

Despite initial prostaglandin infusion and mechanical ventilation, 26 (57.8%) neonates developed severe hypoxemia with oxygen saturation of less than 65% and received urgent BAS within the first 24 h. Among them, two neonates also needed subsequent arterial switch operation within the following 24 h after BAS, because of still inadequate oxygen saturation after BAS. The remaining 24 neonates underwent elective arterial switch operation at an average age of 8 days (range, 4–16 days).

### Non-urgent BAS group

19 (42.2%) neonates had good oxygen saturation initially and did not require urgent BAS, but four of them received elective BAS at the 2nd and 3rd day of life, respectively. Elective arterial switch operation in this group was performed at an average age of 10 days (range, 5–30 days).

The postoperative course in the cardiac intensive care unit was uncomplicated for all but three children. One child developed laryngomalacia, a second one had pulmonary valve stenosis that had to be widened at the age of 2 months, and a third one developed persistent pulmonary hypertension. Median follow-up period in our cohort was 41 months (range, 6 weeks–7 years).

There were no statistically significant differences between the urgent and non-urgent BAS group with regard to maternal age, gestational age at delivery, prevalence of cesarean section, birth weight, Apgar score at the 1st minute of life, and age of life at arterial switch operation (Table 1).

### Predictive reliability of the former prenatal ultrasound reports

In the former prenatal ultrasound reports, 15 fetuses were predicted to have a restrictive FO postnatally and to require urgent BAS. Subsequently, they were delivered by cesarean section. Postnatally, only 11 of them had restrictive FO (73.3% true-positive diagnoses), whereas four had normal FO anatomy and achieved good oxygen saturation contrary to their prenatal prediction and did not require urgent BAS (26.7% false-positive diagnoses). In contrast, 30 fetuses were predicted to have a normal FO anatomy which was confirmed postnatally in only 19 neonates (63.3% true-negative diagnoses), whereas 11 neonates had a restrictive FO contrary to the prenatal prediction and also required urgent BAS (36.7% false-negative diagnoses) (Tables 3 and 4).

**Table 4:** Prediction of FO anatomy and of BAS in former ultrasound reports and after retrospective re-analysis of 19 fetuses that did not underwent urgent BAS

Patient no	Former prenatal ultrasound report	Retrospective re-analysis of FO assessment	Retrospective re-analysis of FO diameter (mm)	Retrospective re-analysis of FO flap movement	Retrospective re-analysis of PV flow pattern	Retrospective re-analysis of PV peak velocity(cm/s)	Retrospective re-analysis of DA flow direction	Delivery Mode	Birth weight (kg)	Postnatal findings	Urgent BAS
1	Restrictive	Normal	8	Normal	Pulsatile	25	Antegr	CESAREAN	2700	FO > 7 mm, normal flap motility	No
2	Restrictive	Normal	10	Normal	Plateau	38	Antegr	Cesarean	4000	FO > 7 mm, normal flap motility	No
3	Normal	Normal	8	Normal	Pulsatile	39	Antegr	Vaginal	3300	FO > 7 mm, normal flap motility	No
4	Normal	Normal	10	Normal	Plateau	25	Antegr	Vaginal	3500	FO > 7 mm, normal flap motility	No
5	Normal	Normal	8	Normal	Plateau	25	Antegr	Vaginal	3900	FO > 7 mm, normal flap motility	No
6	Normal	Normal	9	Normal	Plateau	35	Antegr	Vaginal	3800	FO > 7 mm, normal flap motility	No
7	Normal	Normal	8	Normal	Plateau	30	Antegr	Vaginal	3700	FO > 7 mm, normal flap motility	No
8	Normal	Normal	10	Normal	Plateau	40	Antegr	Cesarean	3500	FO > 7 mm, normal flap motility	No
9	Normal	Normal	10	Normal	Plateau	25	Antegr	Cesarean	3400	FO > 7 mm, normal flap motility	No
10	Normal	Normal	8	Normal	Plateau	30	Antegr	Cesarean	3600	FO > 7 mm, normal flap motility	No
11	Restrictive	Normal	10	Fixed	–	–	Antegr	Cesarean	3000	FO > 7 mm, normal flap motility	No
12	Restrictive	Normal	8	Flat	–	–	Antegr	Cesarean	3600	FO > 7 mm, normal flap motility	No
13	Normal	Normal	8	Normal	–	–	Antegr	Vaginal	3200	FO > 7 mm, normal flap motility	No
14	Normal	Normal	8	Normal	–	–	Antegr	Cesarean	3400	FO > 7 mm, normal flap motility	No
15	Normal	Normal	8	Normal	–	–	Antegr	Vaginal	3300	FO > 7 mm, normal flap motility	No
16	Normal	Normal	8	Normal	–	–	Antegr	CESAREAN	3000	FO > 7 mm, normal flap motility	No
17	Normal	Normal	9	Normal	–	–	Antegr	CESAREAN	3700	FO > 7 mm, normal flap motility	No
18	Normal	Normal	9	Normal	–	–	Antegr	Vaginal	3400	FO > 7 mm, normal flap motility	No
19	Normal	Normal	10	Normal	–	–	Antegr	Vaginal	3500	FO > 7 mm, normal flap motility	No

Overall, restrictive FO was correctly predicted in only 11 of 22 cases in the former prenatal ultrasound reports (sensitivity of 50%) whereas a normal FO size was correctly predicted in 19 of 23 cases (specificity of 83%).

### Establishing reliable diagnostic cardiac parameters

The cardiac parameters, that were retrospectively re-calculated, are shown in Table 2. Overall, the right and left atrial diameters, the prevalence of a redundant (‘spinnaker-like’) appearance of the FO flap, the flow patterns in the PV, the diameter of the DA, and the flow direction through the DA did not differ significantly between both groups.

Significant differences were observed in the FO diameter, the FO flap mobility, and the PV maximum systolic flow velocity: The FO diameter was significantly smaller in the urgent BAS group with a median of 5.7 mm compared with 8.7 mm in the non-urgent BAS group, and a FO diameter of less than 7 mm was highly predictive for restrictive FO (*p* < 0.01). The FO flap mobility was significantly altered with either a fixed appearance in 30.8% of cases of the urgent BAS group compared with 5.3% in the non-urgent BAS group (*p* = 0.035), or a hypermobile flap movement in 26.9% of cases in the urgent BAS group. Because a hypermobile flap was exclusively seen in the urgent BAS group, it was highly predictive for a restrictive FO (*p* = 0.014).

The maximum systolic flow velocities in the PV were also significantly increased in the urgent BAS group with 35 cm/s compared with 31 cm/s in the non-urgent BAS group (*p* = 0.021), but no cut-off value to reliably distinguish between a restrictive and normal FO could be identified.

Thus, the three most significant markers to predict a restrictive FO were the FO diameter of less than 7 mm (*p* < 0.01) and a fixed (*p* = 0.035) or hypermobile FO flap (*p* = 0.014).

### Reliability to correctly predict FO anatomy

After retrospective re-analysis of the FO size and the FO flap mobility, all 22 cases with restrictive FO as well as all 23 cases with normal FO anatomy could correctly be predicted. Both the positive predictive value (PPV) to predict a restrictive FO and the negative predictive value (NPV) to predict normal FO anatomy were 100% each (with a sensitivity and specificity of 100%).

### Reliability to predict the likelihood of urgent BAS in restrictive FO

After retrospective re-assessment, the author correctly predicted the need for urgent BAS in all 22 fetuses with restrictive FO (100% true-positive diagnoses) (Table 3). Among these 22 fetuses, 20 (90.9%) had a FO diameter of less than 7 mm, 8 (36.4%) had a fixed and 7 (31.8%) had a hypermobile FO flap. The maximum systolic flow velocity in the PV was also significantly increased (*p* = 0.021) with a median of 35 cm/s, including four fetuses with a maximum flow velocity of more than 41 cm/s. Overall, the PPV to predict the likelihood of urgent BAS in fetuses with a correctly predicted restrictive FO was 100%.

### Reliability to predict the likelihood of urgent BAS in normal FO anatomy

In addition, the author also correctly predicted the remaining 23 fetuses to have an adequate FO size. As presumed, 19 of the 23 neonates achieved good oxygen saturation and did not require urgent BAS (Table 4). All of them had an adequate FO diameter of more than 7 mm and normal FO flap swinging movements of 30–50°. But the remaining four neonates unexpectedly developed early cyanosis (‘bad mixers’) and also required urgent BAS, although all of them had postnatally confirmed adequate FO diameters of more than 7 mm and normal FO flap swinging movements. In three of these four neonates, the pulmonary veins were also assessed prenatally and also showed a normal plateau-like flow pattern and normal systolic maximum flow velocities of not more than 30 cm/s (Table 3). As this situation cannot be predicted prenatally, the negative predictive value (NPV) for the likelihood of urgent BAS in fetuses with normal FO anatomy was only 82.6% with a false-negative rate (FNR) of 17.8%.

## Discussion

Indisputably, prenatal prediction of a restrictive FO with planned delivery in a tertiary cardiac center and consecutively cardiac intervention within 24 h after birth improves survival, whereas a significant delay of diagnosis and intervention may result in severe and life-threatening neonatal hypoxia. Several prenatal cardiac parameters and classification systems have previously been evaluated to predict premature restriction of the FO and the likelihood of urgent BAS [6, 7, 9, 14–21]. Table 5 shows an overview of the previously published series and the included cardiac parameters.

**Table 5:** Significance of cardiac parameters in fetuses with TGA in previously published studies

	Maeno et al. [14]	Jouannic et al. [15]	Punn et Silverman [16]	Vigneswaran et al. [6]	Tuo et al. [18]	Slodki et al. [19]	Gatta et al. [20]	Patey et al. [7]	Lachaut et al. [9]	Bucca et al. [21]	Gottschalk et al
	1999	2004	2011	2017	2017	2018	2021	2021	2021	2022	2023
	*n* = 16	*n* = 130	*n* = 26	*n* = 40	*n* = 40	*n* = 51	*n* = 60	*n* = 13	*n* = 59	*n* = 292	*n* = 45
Restrictive	s	s	–	s	s	n.s	s	s	s	s	s
FO appearance											
Redundant	n.s	n.s	n.s	s	s	–	s	s	–	n.s	n.s
FO septum											
Flat	n.s	n.s	n.s	s	–	s	n.s	n.s	–	n.s	n.s
FO septum											
Fixed	n.s	n.s	n.s	s	–	–	n.s	n.s	–	n.s	s
FO septum											
Hypermobile FO septum	n.s	–	s	n.s	n.s	-	n.s	n.s	-	s	s
Increased	–	–	–	–	–	s	–	–	–	–	s
PV maximum systolic flow											
Abnormal PV flow pattern	–	–	–	–	n.s	–	–	–	–	–	n.s
Restrictive	s	s	n.s	n.s	n.s	–	n.s	n.s	s	n.s	n.s
DA appearance											
Reversed	–	–	s	–	n.s	n.s	n.s	–	s	n.s	n.s
DA flow											
Reduced	–	–	–	–	s	–	n.s	–	–	s	n.s
RA diameter											
Mean ratio FO/AV diameter	–	–	–	s	–	–	–	–	–	s	–
Mean ratio FO/septal length	–	–	–	s	–	–	–	–	–	s	–
Prevalence of urgent BAS	–	23% BAS	54% BAS	30% BAS	50% BAS	57% BAS	45% BAS	54% BAS	–	–	58% BAS

*s* significant, *n.s.* not significant, – not evaluated

These series showed contradictory reliabilities of the different cardiac parameters in predicting the postnatal FO anatomy and the likelihood of urgent BAS. These difficulties in predicting postnatal hypoxia may be explained by several factors. First, fetal hemodynamic parameters in late pregnancy are influenced by several factors other than those related to FO diameter, such as breathing movements, greater sensitivity to oxygen, and physiological right ventricular preponderance [15]. In addition, all published data relied on retrospective studies and included different cardiac parameters that were obtained at different gestational ages with highly variable time intervals between last prenatal echocardiography and birth. Moreover, postnatal oxygen saturation cut-off values differed, and the indication to perform an urgent BAS was not standardized in previously published reports, making those data only to a limited extend comparable.

At first, Maeno et al. described 16 fetuses with isolated d-TGA in 1999 [14]. They assessed the appearance of the FO and the FO flap as well as the DA diameter, but last sonographic assessment prior to delivery was performed at a mean gestational age of 31 weeks without performing an additional late echocardiography. They concluded that the sensitivity to predict a restrictive FO is low. But in a significant subset of fetuses, FO restriction develops not until the late third trimester and last echocardiography at 31 weeks of gestation may be too early to detect significant restriction of the FO in these cases. As the critical period is near term, we strongly recommend to perform an additional late echocardiography after 35 weeks of gestation in all fetuses with d-TGA to once again re-assess the FO anatomy and to re-evaluate the likelihood of postnatal urgent BAS.

Since then, most recent series including a large meta-analysis by Buca et al. described a significantly smaller FO diameter in fetuses that required urgent BAS [6, 9, 15, 18, 20, 21], but a FO diameter cut-off value to reliably predict a restrictive FO was only described in one single series [20]. In this series, 60 fetuses with isolated d-TGA were retrospectively assessed and the authors concluded that a FO diameter of more than 6.5 mm predicts a normal FO with 100% sensitivity and a false-positive rate of 45%. We could confirm, that a FO diameter of less than 7 mm is highly predictive for a restrictive FO and need for urgent BAS (*p* < 0.01).

Inconsistent findings were published with regard to the septum primum appearance and the FO flap movement. Some authors stated that a redundant, ‘spinnaker-like’ [9, 18, 20, 21] or flat [6, 19] FO flap significantly predicts a restrictive FO, whereas other authors could not confirm this finding [14–16]. Vigneswaran et al. postulated that a redundant FO may be explained as a variant of the normal third trimester atrial septum deviation rather than as a pathological marker [6]. In contrast, a redundant FO flap may cause an occlusion of the FO orifice in some cases as it may mimic primary restriction of FO in cases of an aneurysmal septum primum. Some authors described a hypermobile FO flap as a significant predictor for restrictive FO [16, 21], whereas others could not support this finding and assumed that this may be related to simulated breathing movements or hiccoughs rather than to a restrictive FO [6]. In our series, both a fixed and hypermobile flap mobility were the most significant predictors for a restrictive FO and the need for an urgent BAS (*p* = 0.035 and *p* = 0.014, respectively).

Only one previously published study assessed the flow velocity in the pulmonary veins (PV) and described a significantly increased maximum systolic flow in the PV in restrictive FO. They stated, that a cut-off value of 41 cm/s maximum flow velocity provided maximum specificity of 100% with a positive predictive value of 100% [19]. In accordance, we could confirm that the maximum flow velocity in the PV is increased in restricted FO, but this difference reached no statistical significance (*p* = 0.216) in our data and no cut-off value to reliably predict the restrictive FO could be identified. In addition, the flow pattern in the PV was not significantly altered in fetuses with restrictive FO. Therefore, assessment of PV flow did not improve the reliability of prediction.

Although we were able to correctly predict the postnatal FO anatomy with a sensitivity and specificity of 100% in our re-analysis, the neonates’ ability to achieve a sufficient O_2_ saturation remains unpredictable. Among our 23 neonates with a normal FO size and normal FO flap mobility, 17% were ‘bad mixer’, who postnatally did not achieve a sufficient O_2_ saturation and unexpectedly required urgent BAS also. As this situation escapes prenatal prediction, a normal FO anatomy consequently does not ensure an adequate interatrial shunting in all cases and the negative predictive value to reliably exclude an urgent BAS can naturally never reach 100%. This finding is in accordance with the study by Tuo et al. who described 10 of 40 fetuses that unexpectedly required urgent BAS (false-negative diagnoses in 25%) and who stated that “we should never trust fetuses with d-TGA, even when they seem to have a foramen ovale of normal appearance” [18]. Therefore, all fetuses with prenatally diagnosed d-TGA should always be delivered in a tertiary center with cardiac catheter stand-by allowing BAS within the first 24 h after birth, regardless of their predicted FO anatomy.

The prevalence of urgent BAS within the first 24 h in our cohort was 58%. In previously published prenatal series, the prevalence varied between 23% [15] and 57% [19]. These data are also comparable only to a certain extent because the indication to perform a BAS within the first 24 h was not standardized in different centers and depended on multiple factors including subjective assessment of first neonatal clinical presentation, variable institutional thresholds of oxygen saturation for urgent BAS, variable dosages of applied prostaglandin to prevent ductal closure, or the availability of a permanent cardiac catheter stand-by that allows urgent BAS at any time.

Besides the conventional echocardiographic assessment, very few recent studies described modern techniques on cardiac functional assessment to improve diagnostic accuracy and to optimize perinatal management [7, 24]. Patey and colleagues proposed a novel fetal index with cardiac parameters obtained by spectral pulsed wave tissue-Doppler imaging (PW-TDI) and speckle-tracking echocardiography (STE) and demonstrated significant cardiac geometrical and functional alterations in fetuses with d-TGA that required urgent BAS compared with fetuses that did not. They described the left ventricle rotation-to-shortening ratio, a ratio of apical systolic rotation to basal circumferential strain, as a marker of sub-endocardial dysfunction with the highest sensitivity for prediction of urgent BAS. However, this marker was only evaluated in a very small number of 13 pregnancies and has to be validated in larger prospective trials yet. So far it remains unclear, whether the use of PW-TDI or STE may also improve the prediction of the likelihood for urgent BAS in the subgroup of ‘bad mixer’ fetuses with normal FO size that escapes conventional echocardiography. In addition, calculation of the PW-TDI or STE parameters is more time-consuming, and the specificity of 83% seems to be no higher than the sensitivity of the previously published conventional echocardiographic parameters.

Other few series described the clinical utility of fetal cardiac magnetic resonance imaging (cardiac MRI) as a complementary tool to conventional echocardiography [23, 24, 28, 29]. Although MRI may improve fetal cardiovascular imaging, data on the use of cardiac MRI in prenatal diagnosis of cardiac defects including fetal d-TGA are still missing. Future studies are needed to provide more information on the reliability of fetal cardio MRI in the diagnosis of cardiac defects. Until then, conventional fetal echocardiography remains the ‘gold standard’ in fetal cardiology.

### Limitations of our study

The retrospective nature of our study is the main limitation, and the retrospective offline re-analysis of cardiac parameters may be operator-dependent. Larger prospective series with longitudinal follow-up and standardized postnatal treatment and standardized indications for urgent BAS are needed. The institutional threshold of 65% for pre-ductal oxygen saturation that was selected in our cohort to perform urgent BAS may be questionable, but it is commonly used in clinical practice and in most of the previously published prenatal series.

## Conclusion

In fetuses with d-TGA, conventional fetal echocardiography with assessment of the FO size and FO flap mobility allows a reliable prenatal prediction of both, the restrictive and the normal FO, if fetal echocardiography is performed late in the third trimester within the last 2 weeks before birth. Prediction of the likelihood of urgent BAS also succeeds reliably in all fetuses with restrictive FO, but identification of the small subset of fetuses with normal FO anatomy that also requires urgent BAS (‘bad mixer’) fails, because the ability of sufficient postnatal interatrial mixing cannot be predicted prenatally. Therefore, all fetuses with prenatally diagnosed d-TGA should always be delivered in a tertiary center with cardiac catheter stand-by allowing BAS within the first 24 h after birth, regardless of their predicted FO anatomy.

## Data Availability

All data supporting the findings of this study are available within the article.
